# Impact of *Chlorella vulgaris* and probiotic supplementation on performance, immunity and intestinal microbiota of broiler chickens

**DOI:** 10.1371/journal.pone.0313736

**Published:** 2025-01-27

**Authors:** Verena Pereira Dinalli, Marcio Carvalho Costa, Emerson José Venâncio, João Antônio Barbosa Filho, José Antônio Bessegatto, Augusto Tasch Holkem, Amauri Alcindo Alfieri, Caio Abercio da Silva, Alexandre Oba

**Affiliations:** 1 Department of Animal Science, State University of Londrina (UEL), Londrina, Paraná, Brazil; 2 Department of Biomedical Sciences, University of Montreal, Saint-Hyacinthe, Quebec, Canada; 3 Department of Pathological Sciences, State University of Londrina (UEL), Londrina, Paraná, Brazil; 4 Laboratory of Animal Virology, Department of Veterinary Preventive Medicine, State University of Londrina (UEL), Londrina, Paraná, Brazil; Tokat Gaziosmanpaşa University: Tokat Gaziosmanpasa Universitesi, TÜRKIYE

## Abstract

*Chlorella vulgaris* has antioxidant, antimicrobial, and anti-inflammatory properties, as well as the probiotic that is important for keeping the intestinal microbiota balanced. The objective was to test the impact of supplementation with microalgae and/or probiotics on broiler chickens’ performance, immunity, and intestinal microbiota. The experimental design was in randomized blocks in a 4x2 factorial scheme, with four levels of inclusion of *C*. *vulgaris* (0; 0.25; 0.50 and 1%) associated or not with a commercial probiotic with five replications of 26 chickens per experimental unit. The results showed that probiotics improved feed conversion. Probiotics increased the productivity index only at 0.25% *C*. *vulgaris* supplementation. There was a reduction in spleen weight at 42 days of age in chickens fed with probiotics, but the different treatments did not alter serum antibodies. Sampling age had a significant impact on richness addressed by the number of observed genera and diversity addressed by the Shannon index. The most abundant phylum in the chicken intestinal tract was Firmicutes followed by Bacteroidetes and Proteobacteria. *Bifidobacterium* spp. was found in animals receiving 1% microalgae and probiotics on day 42, suggesting that this genus has benefited from microalgae supplementation. It is concluded that the probiotic and *C*. *vulgaris* have the potential to improve performance without causing major changes in the immunity and cecal microbiota.

## Introduction

The increasing consumption of animal protein concomitant with the restriction on the use of growth promoter antibiotics has led to the search of alternatives to maintain performance in the food animal industry. A healthy gut is critical to the general health and welfare of broiler chickens. In order to have optimal intestinal health, it is necessary that animals have a balanced microbiota that constantly interacts with the host. The composition of the diet is a major factor influencing the microbiota and health of the gastrointestinal tract [1]. Therefore, the intake of dietary supplements, particularly probiotics and prebiotics, can produce beneficial changes in the morphology of the mucosa and in microbiota composition [2, 3].

*Chlorella vulgaris* is a unicellular green microalgae, which stands out for its high biomass production [4], being used as an additive in human and animal food [5]. It is used in the form dried powdered biomass and has about 60% protein [6], 1–4% chlorophyll, 9–18% fiber, vitamins and minerals [7], and polyunsaturated fatty acids [8]. In addition, *Chlorella vulgaris* contributes to the health of chickens by exerting antimicrobial activity [9], presents prebiotic activity [10] and improves the immune response related to inflammatory status. Thus, it can contribute to greater weight gain in broilers, as observed by Roques *et al*. [11] and El-Bahr *et al*. [12] providing 0.8% and 0.1% of *Chlorella vulgaris*, respectively.

Probiotics are live micro-organisms which confer health benefits to the host [13]. Several microbial species such as *Lactobacillus* spp, *Bacillus* spp, *Bifidobacterium* spp, and *Enterococcus* spp have been used as probiotics in the poultry industry [14]. The functionality of multi-strain/multi-species probiotics can be more consistent and more effective than that of a mono-strain. Broilers treated with a multi-species probiotic have increased levels of lysozyme, IgA, T lymphocyte response, of IgA, IgG, and IgM-producing cells in the intestinal mucosa, indicating the potential of these species in stimulating the immune system [15–17].

Supplementation of broiler chickens with microalgae and probiotics has been used in poultry production in an attempt to increase the production of short-chain fatty acids (SCFAs) and to decrease the proliferation of pathogenic microbes by the reduction of the intestinal pH and direct competition for nutrients, thus maintaining the balance necessary to a healthy intestine [18, 19]. In addition, probiotics and microalgae can stimulate different subsets of immune cells to produce cytokines, which in turn play a role in inducing and regulating the immune response [20, 21].

Few studies have evaluated the impact of microalgae and probiotics on the intestinal microbiota of chickens [22, 23]. More data is necessary to allow the establishment of consensus taxa associated with nutritional supplementation and growth performance, since environmental and methodological factors largely vary across studies [24, 25]. In addition, the use of probiotics in production animals remains controversial as studies continue to produce conflicting results [26, 27], mainly because of uncontrolled environmental factors that are likely influencing the efficacy of those supplements, as well as the different microorganisms and doses used as probiotics. This study was designed to test the impact of supplementation with microalgae and/or probiotics on performance, immunity, and the intestinal microbiota of broiler chickens. Based on previous scientific evidences, we hypothesized that supplementation with *Chlorella vulgaris* and probiotics would independently improve immunity, microbiota composition and bird performance. In addition, the hypotheses that a synergistic interaction between the two supplements would occur based on the concept of symbiotic products that provide a prebiotic substratum for probiotic microorganisms.

## Material and methods

### Study design

In this study, one thousand forty 1-day-old male chicks (Cobb 500) were allocated into cages of 2.10 m^2^ (12 animals per m^2^) at the poultry research center of the University of Londrina. This study was approved by the University of Londrina’s Animal Care and Use Committee (process number: 10985.2019.33).

The experimental design was in randomized blocks in a 4x2 factorial scheme, with four levels of inclusion of *C*. *vulgaris* (0; 0.25; 0.50 and 1%) associated or not with a commercial probiotic (0.02% of Probimais^®^ A–Biomart/São Paulo/Brazil) with five replications of 26 chickens per experimental unit. The broiler chickens received 24 hours of light for 14 days, due to the heating system using 250W lamps. After this, the birds received 18 hours of light per day. The average temperature in the experimental period was 26 ± 4 °C. The birds received water and food *ad libitum* during the 42-day experimental period. The commercial probiotic contained *Bacillus subtilis* (3.6x10^9^ CFU/g); *Bifidobacterium bifidum* (2.5x10^9^ CFU/g); *Enterococcus faecium* (2.6x10^9^ CFU/g) and *Lactobacillus acidophilus* (1.3x10^9^ CFU/g). For the formulation of the diets, the nutritional composition of the *C*. *vulgaris* was considered according to Kang *et al*. [5] and probiotic was included as inert. The experimental diets are presented in the supplementary material (S1 Table).

### Performance

For performance analysis, one thousand forty chickens were assessed at 7, 21, 35 and 42 days of age. Feed intake (FI) was determined by calculating the difference between the weight of the feed offered and the weight of the leftover feed, the difference was divided by the number of birds. Weight gain (WG) was calculated as the final weight minus the initial weight of the birds and feed conversion (FC) was calculated as the total amount of feed consumed divided by the total weight gain. These data were adjusted for the number and weight of birds killed during the experimental period. Viability (V) was calculated with the following formula: (100—mortality in percent). Production efficiency index (PEI) was calculated using the following formula: PEI = (daily weight gain (kg) × viability /feed conversion ratio) × 100, according to the method described previously by Lorençon *et al*. [28].

### Immunological parameters

For immunological analysis, a total of 80 chickens were used (n = 10/treatment), were weighed and slaughtered through electrical stunning at 21 and 43 days of age [29]. The chickens were necropsied for the collection of spleen and bursa of Fabricius, which were weighed and values were expressed as a percentage of the live weight of the bird [30]. Blood samples from two chickens per experimental unit (n = 10/treatment) were collected at 13 days, 21 days, and 42 days of age. The serum was stored at -20 °C until use.

To evaluate the humoral immune response, two chickens from each experimental unit (n = 10/treatment) were inoculated intramuscularly with 5% sheep red blood cells (SRBC) in phosphate-saline buffer (1X PBS) pH 7.2 (200 μL) on the 14^th^ and 35^th^ days of the experiment. Serological analyzes were performed with serum at 21 and 42 days of age.

The analysis of anti-SRBC antibody levels were performed using ELISA as described by Silva *et al*. [31] with the following modifications. SRBC protein extract was used at 5 μg/mL, the dilution of serum was 1:80 in 1X PBS with 1% non-fat dry milk (1 X PBS/1% milk), and the dilution of HRP antibodies was 1:25000 in 1X PBS/1% milk. To determine the levels of natural anti-KLH antibodies, serum from chickens at 13, 21 and 42 days of age were used, and the ELISA was performed as described above with the following modifications. The microplates were sensitized with KLH (Keyhole limpet hemocyanin at 10 μg/mL) and serum samples were diluted 1:40.

### Microbiota analysis

For microbiota investigation, a total of 40 chickens were used (n = 5/treatment) were electrically stunned in a water bath (Model FX 2.0 Fluxo, Chapecó, Brazil) and then bled before slaughter on day 21 and 42. The animals had their cecal content collected in sterile plastic tubes that were immediately refrigerated (for a maximum of 1.5 hours) and frozen at -80°C until DNA extraction.

The V4 region of the bacterial 16S rRNA gene was amplified by PCR using the following primers: 515F (GTGCCAGCMGCCGCGGTAA) and 806R (GGACTACHVGGGTWTCTAAT) as previously recommended [32]. Sequencing was performed using an Illumina MiSeq platform, using the V2 reagent kit (2 × 250 cycles) at the Genome Quebec Innovation Centre.

Sequence data were processed using the software mothur [33] following the Standard Operating Procedure previously described Kozich *et al*. [34]. Good quality reads were clustered in operation taxonomic units (OTUs) with 97% similarity and classified according to the Ribosomal Database Project databank. OTUs belonging to the same genus were then clustered into “phylotypes” for alpha and beta diversity analyses. The number of observed genera and the Shannon index were used for characterization of alpha diversity. Beta diversity evaluating similarities in composition among samples was addressed by the Jaccard index and the Yue and Clayton indices to compare, respectively, community composition (that considers only presence or absence of the different bacteria) and structure (that considers the different taxa and their proportions within each sample). Beta diversity was explored visually using principal coordinate analysis (PCoA).

### Statistical analysis

For performance and weight of lymphoid organs variables, statistical analysis was performed using analysis of variance for a 4 x 2 factorial model using the software R [35]. When significance was observed (P<0.05), the data was subjected to regression analysis to obtain the model with the best adjustment using the ExpDest.pt package.

The concentrations of IgY, IgM, and IgA were analyzed using the repeated measures procedure in the General Linear Model (GLM), followed by a Tukey post-hoc test of Statistica for Windows (Statsoft 13.0). A P value of 0.05 was used to assess significance among means. The model included microalgae levels, probiotic presence, day, and block as fixed factors, as well as the two-way interactions between microalgae levels and probiotic presence, microalgae levels and day, and the three-way interaction between microalgae levels, day, and probiotic presence, as shown in the following equation:

$$Yijkm=\mu +\alpha i+\beta j+(\alpha \beta )ij+\pi km\left(i\right)+(\alpha \gamma )ik+(\beta \gamma )jk+(\pi \gamma )km\left(i\right)+(\alpha \beta \gamma )ijk+\varepsilon ijkm$$

Where *μ* is the overall mean, *αi* is the effect of the *i*-th level of factor A, *βj* is the effect of the *j*-th level of factor B, (*αβ*)*ij* is the interaction effect of factors A and B, *πkm*(*i*) is the effect of the *m*-th experimental unit within the *i*-th level of factor A and *j*-th level of factor B (Error 1), *γk* is the effect of the *k*-th level of factor C, (*αγ*)*ik* is the interaction effect of factors A and C, (*βγ*)*jk* is the interaction effect of factors B and C, (*πγ*)*km*(*i*) is the interaction effect of the experimental unit and factor C, (*αβγ*)*ijk* is the interaction effect of factors A, B, and C, and *εijkm* is the error term which represents the interaction between factor C (repeated factor) and Error 1 (Error 2).

For microbiota analyses the indices of alpha diversity were compared using a 2-way ANOVA considering time of sampling and treatment (*C*. *vulgaris* and probiotic) as variables. Beta diversity (community composition and structure) was compared using the analysis of molecular variance (AMOVA) test, considering a *P* < 0.05 as significant. Finally, the Linear Discriminant Analysis Effect Size (LEfSe) analysis was used to identify bacterial taxa associated with each experimental group, as well as sampling time [36].

## Results and discussion

### Performance

The treatments did not influence the results obtained for feed intake, weight gain and viability (Table 1). The reasons why viability was low in the experiment is related to the high sanitary challenge and the non-addition of antibiotics in the ration to avoid another variable in the experiment, as this could affect probiotic viability, as well as the utilization of the microalgae in the chicken’s gut. This resulted in a high rate of low development birds, which were removed from the experiment, providing a low viability.

**Table 1:** Mean values referring to feed intake (FI), weight gain (WG), feed conversion (FC), viability (V) and productive efficiency index (PEI) of broilers fed with different levels of *C*. *vulgaris* associated or not to the probiotic.

Parameters (%)	*C*. *vulgaris*				Probiotic			*P*-value		
	0%	0.25%	0.50%	1.00%	without	with	CV (%)	*C*.*vulgaris*	Probiotic	*C*.*vulgaris* x Probiotic
FI (kg/bird)	4.92	4.91	4.94	4.89	4.91	4.92	2.63	0.843	0.842	0.058
WG (kg/bird)	2.84	2.82	2.81	2.81	2.80	2.84	2.72	0.800	0.100	0.088
FC (kg/kg)	1.73	1.74	1.76	1.74	1.76	1.73	1.68	0.419	0.023	0.978
V (%)	81.15	81.92	81.92	84.62	82.31	82.50	7.97	0.662	0.927	0.276
PEI	316.37	315.41	312.16	324.46	312.27	321.93	6.84	0.486	0.429	0.032

*C*. *vulgaris* treatment: regression analysis (*P* < 0.05). Probiotic treatment: F test of comparison of means at a level of 5% significance. CV = Coefficient of Variation.

The feed conversion was enhanced by probiotic addition and an interaction between *C*. *vulgaris* and probiotic was found for PEI (Table 2), in which the addition of only 0.25% of *C*. *vulgaris* with the probiotic provided the best PEI (*P* < 0.05). Elements present in *C*. *vulgaris* such as chlorellin, fibers, polysaccharides, mannan oligosaccharide, rhamnose, galactose, glucose, xylose, arabinose, and mannose can be digested by intestinal microorganisms increasing the proliferation of beneficial bacteria in the gastrointestinal tract, increasing absorption of nutrients [37] in consequence of their higher capacity to extract nutrients from food or of the production of molecules improving intestinal health (e.g. butyrate) by improving tight junctions and production of mucous. In addition, the microalgae are rich in essential polyunsaturated fatty acids, with high levels of α-linolenic acid (omega 3 series fatty acid), which can reduce the formation of eicosanoids, and therefore inflammation [38, 39].

**Table 2:** Deployment of the interaction between the levels of inclusion of *C*. *vulgaris* associated or not with the probiotic for productive efficiency index (PEI) from one to 42 days of age.

*C*. *vulgaris*	Without probiotic	With probiotic	P value
0 (%)	328.93	323.24	0.809
0.25 (%)	299.87b	330.94a	0.032
0.50 (%)	325.27	299.05	0.068
1.00 (%)	314.41	334.51	0.157
P value	0.227	0.067	

Means with significant differences were determined by the F test (P<0.05)

Although the activation of important immune responses consumes nutrients and energy [40], the immune activation achieved by the addition of probiotics is in general associated with better food conversion, possibly by inhibiting sub-clinical infections with pathogens [41], preserving intestinal integrity [42] and increasing the activity of digestive enzymes such as lipase, protease and amylase [43].

In this study, the use of 0.25% *C*. *vulgaris* and probiotic (*P* < 0.05) showed a better productive efficiency index. Several prebiotic substances present in *C*. *vulgaris* could be working as selective substrates for beneficial bacteria [44] because they are resistant to the enzymes of the upper gastrointestinal tract and fermented by the intestinal microbiota in the large intestine [45]. Thus, *C*. *vulgaris* might have enhanced the probiotic strains, which exerts beneficial effects on animal performance. The reasons why this effect was observed only at a level of 0.25% of microalgae is uncertain, but it might be related with the excess of non-digestible carbohydrates stimulating other members of the microbiota. Further *in vitro* or *ex vivo* studies including mechanistic approaches designed to better understand the symbiotic interaction between the two products, as well as the metabolism of this probiotic strain could provide more insights into our current understanding.

### Immunology

The inclusion of *C*. *vulgaris* and/or probiotic in the diet of broilers had no impact on the weight of spleen and bursa of Fabricius (Table 3) at 21 days of age as showed earlier [46]. The addition of probiotic provided a lower (18%) relative weight of the spleen (*P* < 0.05), without changing the relative weight of the bursa of Fabricius at 43 days of age. These results support the capacity of probiotics to enhance intestinal integrity and immunity reducing challenge exposure, avoiding hypertrophy, as the spleen performs the function of capturing antigens, stimulating B lymphocytes and T cells to develop nonspecific and general immunity [47, 48].

**Table 3:** Relative weight of spleen and bursa of Fabricius of broilers fed diets containing *C*.*vulgaris* and/or probiotic inclusion.

Parameters (%)	*C*.*vulgaris*				Probiotic			*P*-value		
	0%	0.25%	0.50%	1.00%	without	with	CV (%)	*C*.*vulgaris*	Probiotic	*C*.*vulgaris* x Probiotic
21 days of the life										
Spleen	0.14	0.13	0.14	0.15	0.14	0.14	18.54	0.221	0.881	0.200
Bursa of Fabricius	0.22	0.22	0.22	0.21	0.21	0.22	22.37	0.825	0.716	0.611
43 days of the life										
Spleen	0.10	0.10	0.10	0.10	0.11	0.09	16.78	0.927	0.036	0.722
Bursa of Fabricius	0.16	0.15	0.16	0.15	0.16	0.15	18.76	0.551	0.581	0.480

*C*. *vulgaris* treatment: regression analysis (*P* < 0.05). Probiotic treatment: F test of comparison of means at a level of 5% significance. CV = Coefficient of Variation.

The results show that the addition of *C*. *vulgaris* and/or probiotics to the diet of broiler chickens did not influence the natural anti-KLH antibodies evaluated at 13, 21 and 42 days of age and anti-SRBC at 21 and 41 days of age (Table 4). These results are consistent with a previous study that did not observe an effect of *C*. *vulgaris* on the production of specific antibodies against vaccine antigens [46]. On other hand, it is interesting to note that some studies have observed an effect of *C*. *vulgaris* on the humoral immune response. These studies show an effect of *C*. *vulgaris* on the concentration of mucosal IgA (20) an serum IgM and IgG [46]. These microalgae can modulate the immune system by having high levels of omega-3 fatty acids, vitamin B12 [49], antioxidants [50] and phenolic compounds [51]. In the present study, no differences were observed in serum immunoglobulins in chickens fed *C*. *vulgaris*, despite similar levels of inclusion in the diets. This may be due to the composition of *C*. *vulgaris*, which was not analyzed to verify the quantify the presence of immunomodulatory components.

**Table 4:** Natural antibodies (13, 21 and 42 days of age) and anti-SRBC- (21 and 42 days of age) in serum of chickens fed diets containing *C*.*vulgaris* and/or probiotic inclusion.

Parameters	*C*.*vulgaris*				Probiotic			*P*-value			
	0%	0.25%	0.50%	1.00%	without	With	CV (%)	*C*.*vulgaris*	Probiotic	*C*.*vulgaris* X Probiotic
Natural antibody titer										
IgY										
IgY 13d	0.02	0.04	0.04	0.06	0.04	0.04	2.28	0.9716	0.3134	0.9239
IgY 21d	0.10	0.11	0.09	0.08	0.10	0.09	4.56			
IgY 42d	0.33	0.30	0.33	0.34	0.35	0.30	8.88			
IgM										
IgM 13d	0.03	0.04	0.04	0.04	0.04	0.04	2.69	0.5332	0.5375	0.4368
IgM 21d	0.13	0.14	0.12	0.12	0.13	0.12	4.50			
IgM 42d	0.23	0.25	0.28	0.30	0.28	0.25	5.62			
IgA										
IgA 13d	0.03	0.02	0.02	0.05	0.02	0.04	2.89	0.1379	0.4236	0.9026
IgA 21d	0.34	0.19	0.18	0.26	0.29	0.19	14.99			
IgA 42d	0.76	0.47	0.56	0.56	0.60	0.57	16.42			
Sheep anti-red blood cell antibody titer										
IgY										
IgY 21d	0.12	0.15	0.13	0.10	0.11	0.13	15.58	0.6144	0.7116	0.2377
IgY 42d	0.42	0.43	0.28	0.48	0.41	0.39	17.27			
IgM										
IgM 21d	0.03	0.003	0.008	0.002	0.02	0.002	4.64	0.8502	0.6124	0.4430
IgM 42d	0.61	0.56	0.57	0.66	0.62	0.59	11.63			
IgA										
IgA 21d	0.06	0.02	0.01	0.01	0.05	0.03	8.79	0.2972	0.7800	0.9440
IgA 42d	0.60	0.30	0.69	0.60	0.49	0.61	22.27			

Differences between the treatment groups are statistically different at P<0.05. CV = Coefficient of Variation.

The addition of probiotic did not change the serum immunoglobulins, similar to the results obtained by Mountzouris et al. [52]. These results are contrary to those obtained by Hassanpour et al. [53] who report that probiotics stimulate immune function and increase the production of immunoglobulins.

### Microbiota analysis

Average and standard deviation of alpha diversity indices found in the cecal microbiota of broiler chickens at two different ages are presented. Sampling age had a significant impact on richness addressed by the number of observed genera on day 21 (68.82 ± 5) and day 42 (2.23 ± 0.30) (*P* < 0.001) and diversity addressed by the Shannon index on day (53.00 ± 5) and day 42 (2.09 ± 0.34) (*P* = 0.031). In this study, age independently modulated the cecal microbiota, decreasing richness and diversity over time, which is in agreement with previous studies [25, 54]. Chicken age is one of the most important factors influencing gastrointestinal bacterial composition, cell density and metabolic function [55]. No differences in alpha diversity indices were observed among treatments.

**Fig 1:**
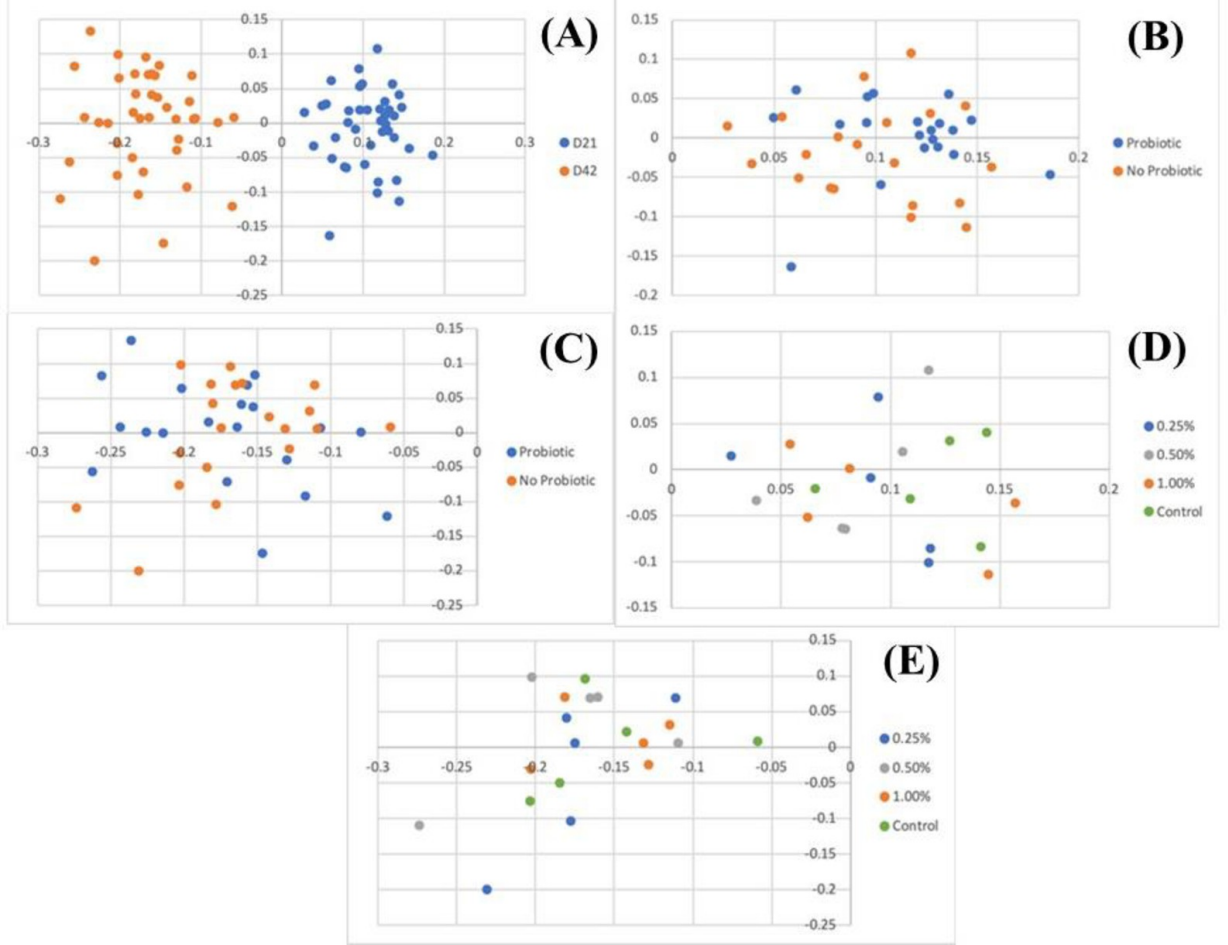
Microbiota similarity represented by PCoA of bacterial composition influenced by age (A), supplementation with probiotic on D21 (B), with probiotic on Day 42 (C), with *C*. *vulgaris* on Day 21 (D) and with *C*. *vulgaris* on Day 42 (E).

**Fig 2:**
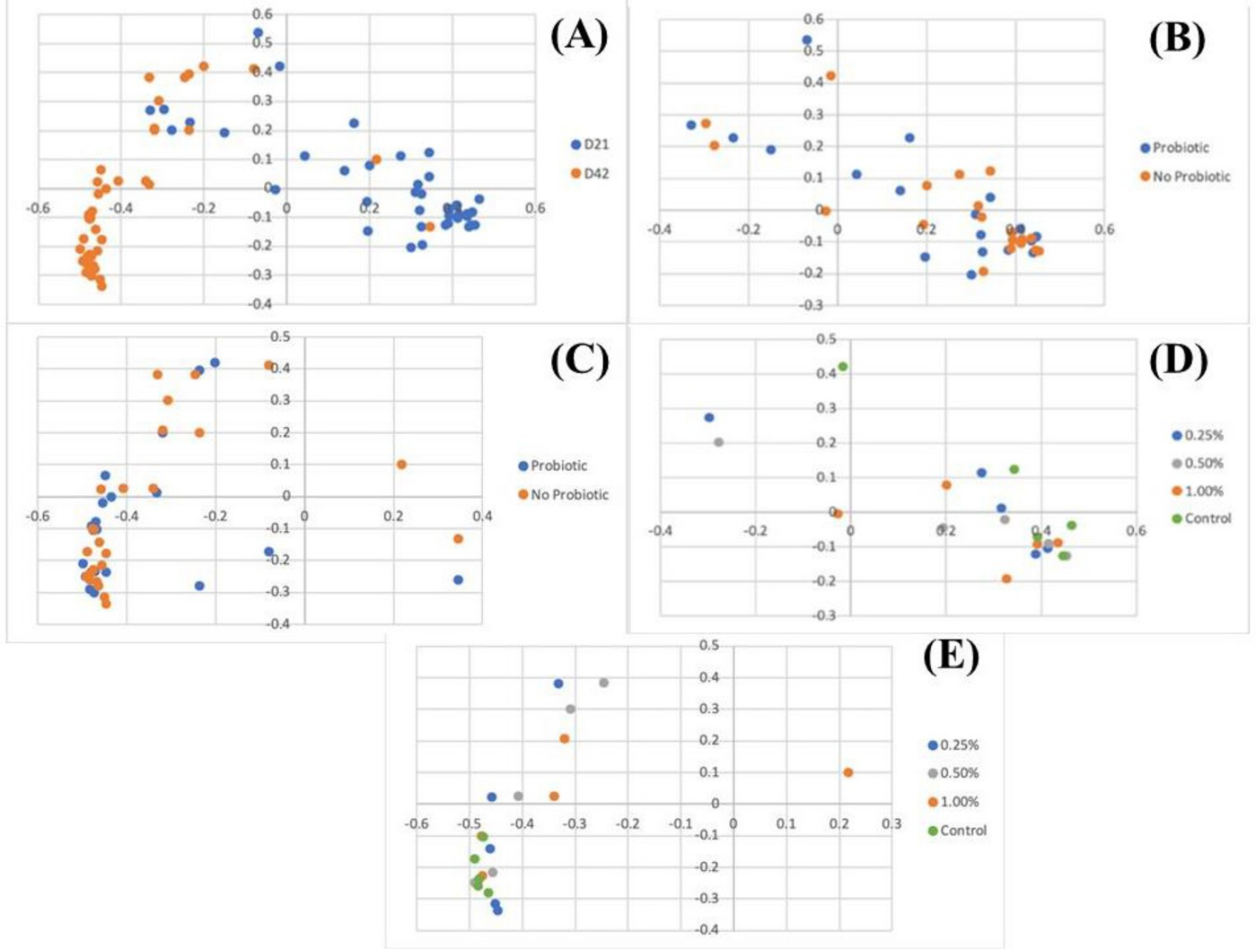
Microbiota similarity represented by PCoA of bacterial structure influenced by age (A), supplementation with probiotic on Day 21 (B), with probiotic on Day 42 (C), with *C*. *vulgaris* on Day 21 (D) and with *C*. *vulgaris* on Day 42 (E).

The similarity between bacterial communities present in each sample is represented by the Principal Coordinate Analysis (PCoA) in Figs 1 and 2 and Table 5. Age had a strong impact in composition and structure, whereas treatment with microalgae or probiotics did not significantly affect community composition or structure. Although not statistically significant, a clustering in microbiota composition of chickens receiving probiotics at Day 21 can be visualized in the PCoA plot (Fig 1B). The lack of significance was likely caused by the presence of one outlier as well as the low sample size, but this might support the fact that modulation of the intestinal microbiota is easier at younger ages [56, 57].

**Table 5:** *P* values of AMOVA test (Statistical comparison of beta diversity).

	Composition	Structure
D21 vs. D42	<0.001	<0.001
Probiotic vs. No probiotic D21	0.250	0.814
Probiotic vs. No probiotic D42	0.982	0.717

Treatment with probiotics and microalgae did not cause significant changes in the structure of the cecal community compared to the control group (AMOVA, *P* > 0.05). In broiler chickens, the administration of microalgae has been associated with prebiotic properties and an increase in the intestinal population of lactic acid bacteria (LAB) [58, 59]. Unlike these earlier studies, treatment with *C*. *vulgaris* and probiotics in the present study did not affect those specific populations. The reason for these divergent data remains unclear, but the distinct levels and the nutritional qualities of *C*. *vulgaris* flour as well as environmental conditions may be responsible. Our data corroborates with the study carried out by Sugiharto and Lauridsen [20] reporting that supplementation of broilers with microalgae (*S*. *platensis*) did not affect the ileal and caecal populations of LAB. Another possible reason for the lack of change in the structure of the cecal community is that probiotics generally do not reduce the total amount or activity of bacteria in the gut, but they can sometimes increase concentrations of bacterial metabolites in broilers chickens [60]. In fact, this is supported by the statistically greater concentration of α-linolenic acid observed in meat of microalgae supplemented animals published somewhere else [61], suggesting either greater production or higher intestinal absorption of those molecules. Further studies using metabolomics approach in the cecal content are required to confirm this.

It has been shown that consequences of early life manipulation of the intestinal microbiota can persist into adulthood, possibly decreasing the incidence of diseases [62–64] and administration of *Lactobacillus reuteri* to broilers during the first week of the life had a positive effect on gut microbiota composition (diversity, abundance, and reduction of pathogens) for up to 6 weeks [65], however in the present work this was not observed.

**Fig 3:**
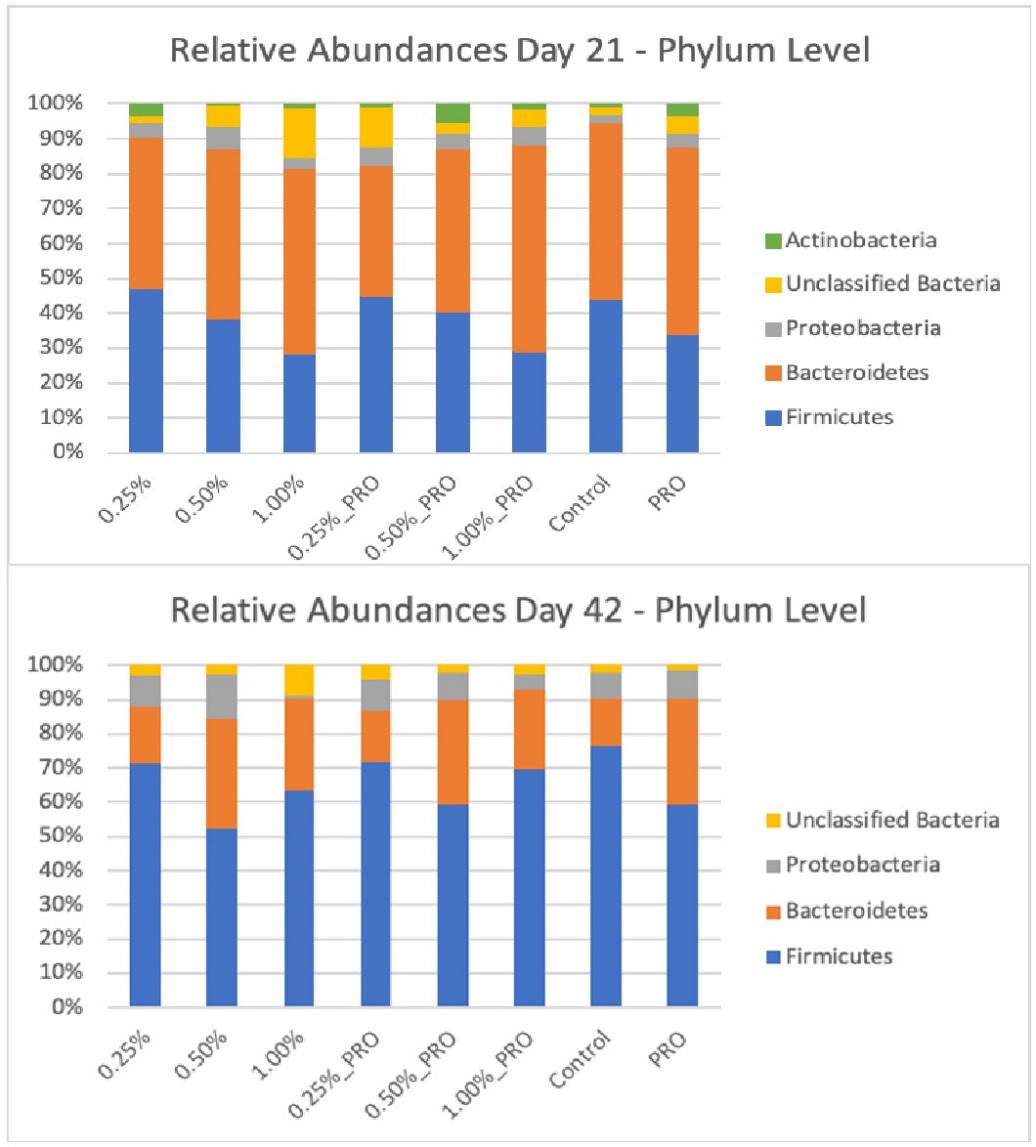
Relative abundances (> 1%) of the main bacterial phyla found in the cecum of broiler chickens treated with microalgae and probiotic and in a control group at two different ages.

**Fig 4:**
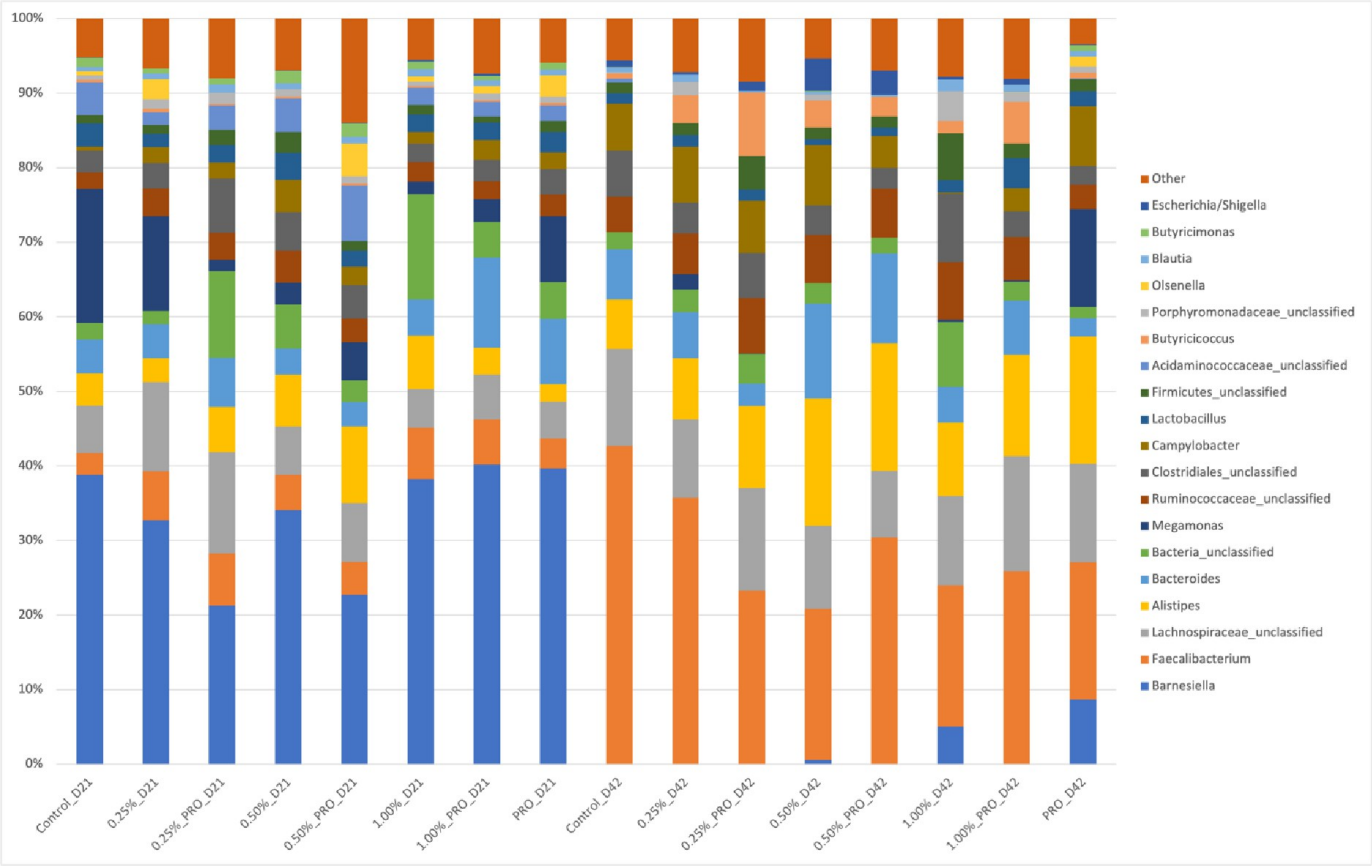
Relative abundances (>1%) of the main bacterial genera found in the cecum of broiler chickens on Day 21 and Day 42 of life.

The relative abundances of different phyla and genus across treatment groups are presented in Figs 3 and 4, respectively. The most abundant phylum in the chicken intestinal tract was Firmicutes followed by Bacteroidetes and Proteobacteria. In addition, members of the Actinobacteria phylum were found in very low abundance in broiler chicken at 21 days of life. At the genus level, *Barnesiella* and *Faecalibacterium* were the most abundant, respectively at 21 days and 42 days of age. The results obtained in this study corroborate with others describing the cecal microbiota of chickens [66–68]. In the study by Oakley and Kogut [69], it was also observed that after the age of 3 weeks, the bacterial population of chickens changed from Proteobacteria, Bacteroides and Firmicutes to mainly Firmicutes.

**Fig 5:**
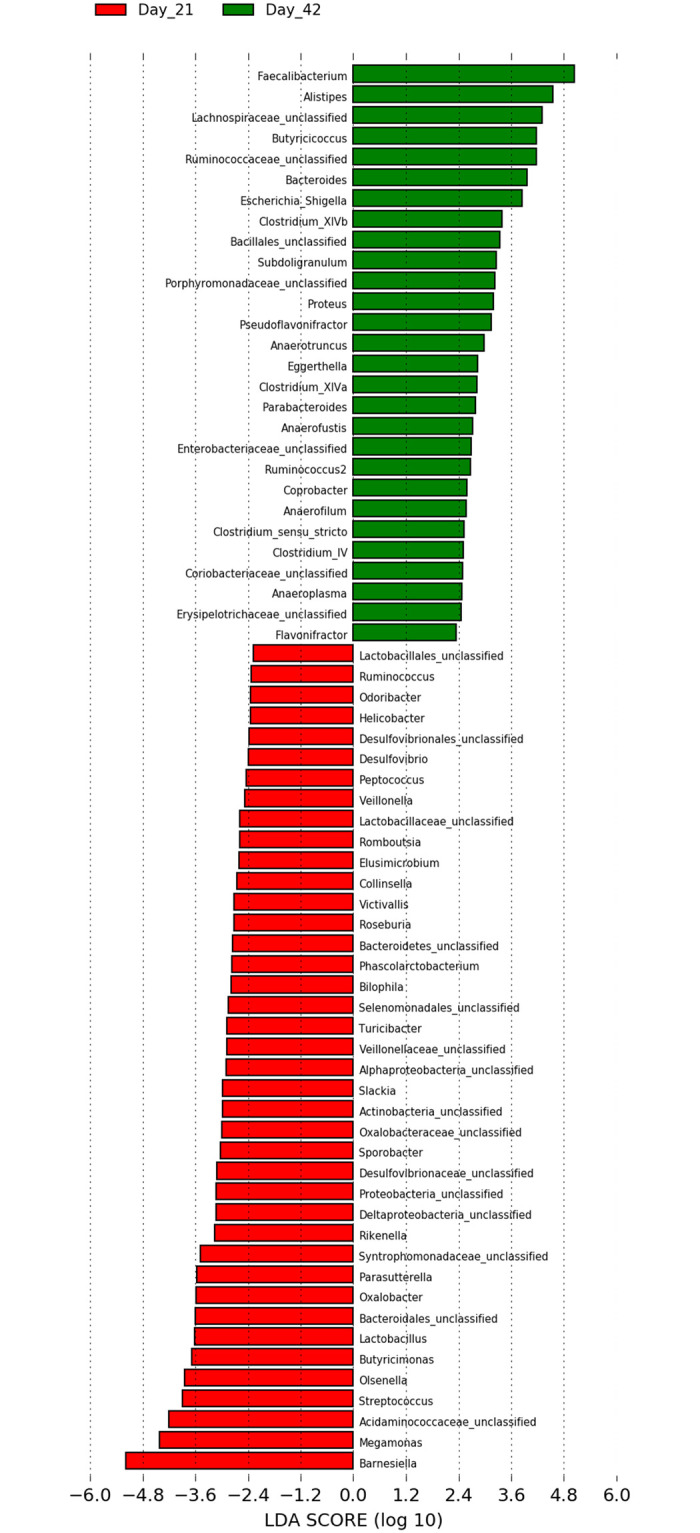
LEfSe analysis representing cecal bacteria that were statistically overrepresented on Day 21 and Day 42 of life in broiler chickens.

**Fig 6:**
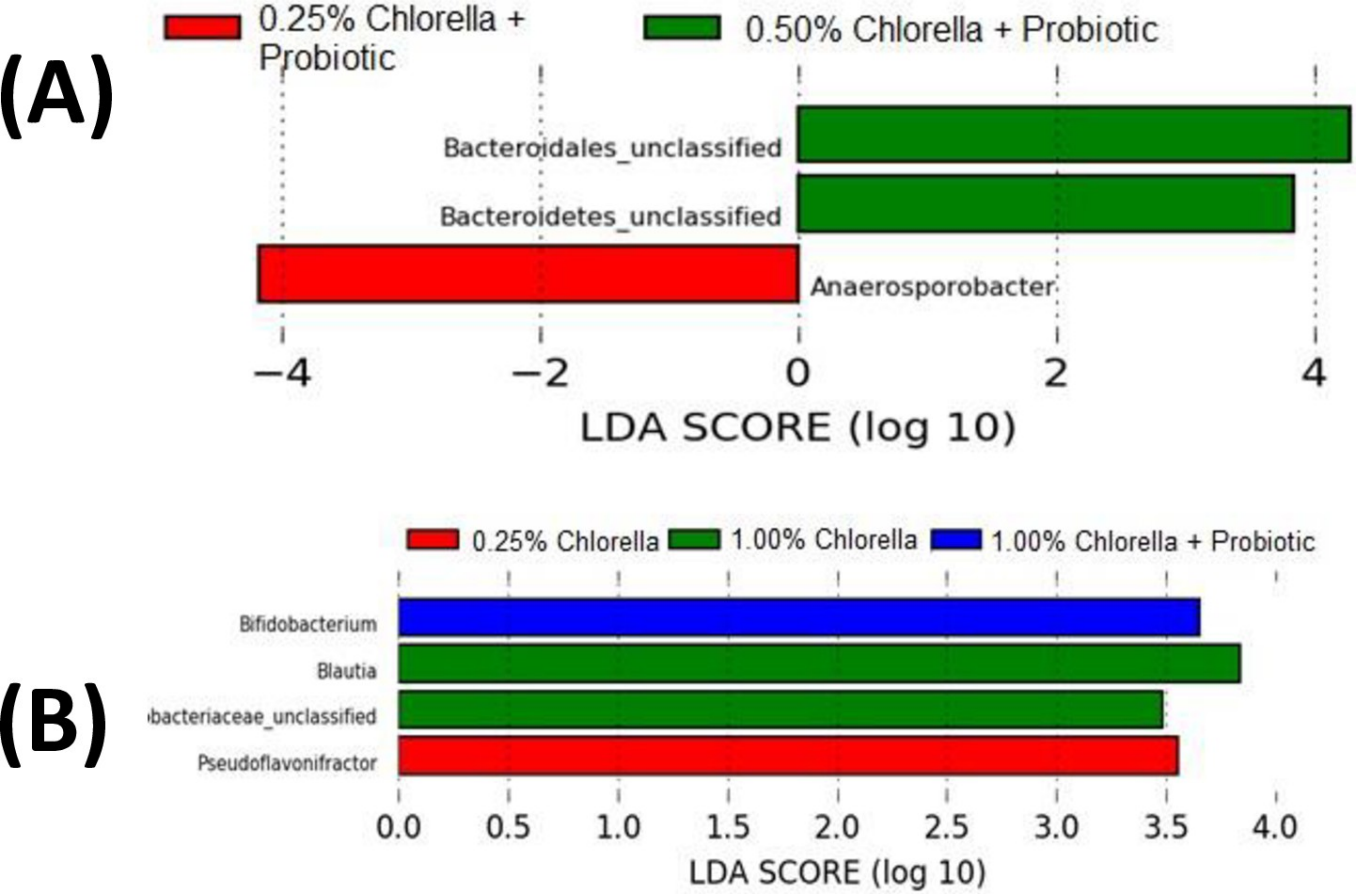
LEfSe analysis representing cecal bacterial that were statistically increased by supplementation with nutritional supplements on Day 21 (A) and Day 42 (B) of life in broiler chickens.

Results of LEfSe analysis are shown in Figs 5 and 6. Several taxa were statistically associated with different ages (D21 and D42 of life). *Faecalibacterium* spp. and *Barnesiella* spp were the most strongly represented in samples collected on day 21 and 42, respectively (Fig 5).

Animals that received 1% microalgae and probiotics on day 42 had statistically greater abundances of *Bifidobacterium* spp. (Fig 6B), suggesting that this genus has benefited from microalgae supplementation likely because of its prebiotic effect. Microalgae species have complex polysaccharides and phytochemicals such as polyphenols, carotenoids, and fatty acids with potential to modulate the microbiota. Polysaccharides from *Grateloupia filicina* and *Eucheuma spinosum* have been shown to significantly promote the proliferation of *Bifidobacterium* [70]. Those findings can be useful in future studies identifying potential prebiotics related to better performance in the search of alternatives to replace the use of growth promoter antibiotics.

## Conclusions

The results of this exploratory investigation support the view that supplementation with *C*. *vulgaris* and probiotics has the potential to improve performance without causing major changes in the cecal microbiota and serum antibodies of broiler chickens. This could be attributed to the capacity of probiotics to enhance intestinal integrity reducing challenge exposure. The present study supports further investigation to confirm this strategy as an alternative to growth promoter antibiotics.

## Supporting information

S1 Table Percentage and calculated composition of the experimental diets in the different stages of creation. (DOCX)
