# Supplementary material for: Impact of Chlorella vulgaris and probiotic supplementation on performance, immunity and intestinal microbiota of broiler chickens
Source: PLoS One. 2025 Jan 27;20(1):e0313736. doi: 10.1371/journal.pone.0313736 (PMC11771937; doi:10.1371/journal.pone.0313736)
Supplement: S1 Table — (DOCX) [file pone.0313736.s001.docx]

Table S1. Percentage and calculated composition of the experimental diets in the different stages of creation.

| Stages of creation | pre-initial (1-7 days) | | | | | Initial (8 – 21 days) | | | | Growth (22 – 35 days) | | | | Termination (36 – 42 days) | | | | |
| --- | --- | --- | --- | --- | --- | --- | --- | --- | --- | --- | --- | --- | --- | --- | --- | --- | --- | --- |
| *C. vulgaris* levels (%) | 0 | 0,25 | 0,50 | 1 | 0 | | 0,25 | 0,50 | 1 | 0 | 0,25 | 0,50 | 1 | 0 | 0,25 | 0,50 | 1 | |
| Ingredients | | | | | | | | | | | | | | | | | |  |
| corn grain | 51,69 | 51,89 | 52,09 | 52,48 | 53,94 | | 54,13 | 54,33 | 54,72 | 61,44 | 61,64 | 61,83 | 62,23 | 67,90 | 68,10 | 68,29 | 68,69 | |
| Soybean meal 46% | 41,98 | 41,62 | 41,25 | 40,53 | 39,50 | | 39,14 | 38,77 | 38,04 | 32,14 | 31,78 | 31,41 | 30,68 | 26,61 | 26,25 | 25,88 | 25,15 | |
| Limestone | 0,94 | 0,95 | 0,96 | 0,98 | 0,86 | | 0,87 | 0,88 | 0,90 | 0,74 | 0,75 | 0,76 | 0,78 | 0,71 | 0,72 | 0,73 | 0,75 | |
| Dicalcium phosphate | 1,80 | 1,79 | 1,78 | 1,76 | 1,58 | | 1,57 | 1,56 | 1,54 | 1,39 | 1,38 | 1,37 | 1,35 | 1,00 | 0,99 | 0,98 | 0,96 | |
| Soy oil | 2,09 | 2,01 | 1,92 | 1,76 | 2,75 | | 2,67 | 2,59 | 2,42 | 2,96 | 2,88 | 2,80 | 2,63 | 2,53 | 2,45 | 2,37 | 2,20 | |
| Salt | 0,53 | 0,53 | 0,53 | 0,53 | 0,51 | | 0,51 | 0,51 | 0,51 | 0,48 | 0,48 | 0,48 | 0,48 | 0,45 | 0,45 | 0,45 | 0,45 | |
| ^1^vitamin supplement | 0,50 | 0,50 | 0,50 | 0,50 | 0,40 | | 0,40 | 0,40 | 0,40 | 0,35 | 0,35 | 0,35 | 0,35 | 0,30 | 0,30 | 0,30 | 0,30 | |
| DL-Methionine | 0,25 | 0,24 | 0,24 | 0,24 | 0,23 | | 0,23 | 0,23 | 0,23 | 0,21 | 0,21 | 0,20 | 0,20 | 0,18 | 0,18 | 0,18 | 0,18 | |
| L-Lysine HCL | 0,14 | 0,13 | 0,13 | 0,13 | 0,15 | | 0,15 | 0,14 | 0,14 | 0,21 | 0,21 | 0,20 | 0,20 | 0,24 | 0,23 | 0,23 | 0,22 | |
| L-Threonine | 0,06 | 0,07 | 0,07 | 0,08 | 0,06 | | 0,07 | 0,07 | 0,08 | 0,07 | 0,07 | 0,08 | 0,09 | 0,06 | 0,07 | 0,08 | 0,09 | |
| *C. vulgaris* | 0,00 | 0,25 | 0,50 | 1,00 | 0,00 | | 0,25 | 0,50 | 1,00 | 0,00 | 0,25 | 0,50 | 1,00 | 0,00 | 0,25 | 0,50 | 1,00 | |
| Inert/probiotic | 0,02 | 0,02 | 0,02 | 0,02 | 0,02 | | 0,02 | 0,02 | 0,02 | 0,02 | 0,02 | 0,02 | 0,02 | 0,02 | 0,02 | 0,02 | 0,02 | |
| Total (kg) | 100 | 100 | 100 | 100 | 100 | | 100 | 100 | 100 | 100 | 100 | 100 | 100 | 100 | 100 | 100 | 100 | |
| Calculated nutritional composition | | | | | | | | | | | | | | | | | |  |
| Calcium (%) | 0,97 | 0,97 | 0,97 | 0,97 | 0,88 | | 0,88 | 0,88 | 0,88 | 0,76 | 0,76 | 0,76 | 0,76 | 0,63 | 0,63 | 0,63 | 0,63 | |
| metabolic energy (kcal/kg) | 2,975 | 2,975 | 2,975 | 2,975 | 3,050 | | 3,050 | 3,050 | 3,050 | 3,150 | 3,150 | 3,150 | 3,150 | 3,200 | 3,200 | 3,200 | 3,200 | |
| Phosphorus available (%) | 0,46 | 0,46 | 0,46 | 0,46 | 0,42 | | 0,42 | 0,42 | 0,42 | 0,37 | 0,37 | 0,37 | 0,37 | 0,30 | 0,30 | 0,30 | 0,30 | |
| total lysine (%) | 1,44 | 1,44 | 1,44 | 1,44 | 1,38 | | 1,38 | 1,38 | 1,38 | 1,24 | 1,24 | 1,24 | 1,24 | 1,12 | 1,12 | 1,12 | 1,12 | |
| total methionine (%) | 0,59 | 0,59 | 0,59 | 0,59 | 0,57 | | 0,57 | 0,57 | 0,57 | 0,51 | 0,51 | 0,51 | 0,51 | 0,46 | 0,46 | 0,46 | 0,46 | |
| total threonine (%) | 0,99 | 0,99 | 0,99 | 0,99 | 0,96 | | 0,96 | 0,96 | 0,96 | 0,86 | 0,86 | 0,86 | 0,86 | 0,77 | 0,77 | 0,77 | 0,77 | |
| crude protein (%) | 24,27 | 24,27 | 24,27 | 24,27 | 23,31 | | 23,31 | 23,31 | 23,31 | 20,58 | 20,58 | 20,58 | 20,58 | 18,57 | 18,57 | 18,57 | 18,57 | |
| Sodium (%) | 0,23 | 0,23 | 0,23 | 0,23 | 0,22 | | 0,22 | 0,22 | 0,22 | 0,21 | 0,21 | 0,21 | 0,21 | 0,20 | 0,20 | 0,20 | 0,20 | |

^1^Vitamin supplement +mineral, amount/kg of feed: vit. A - 2,758,000UI/kg; vit. E – 689,000UI/kg; vit. B1 – 608mg/kg; vit. B2 – 1,655mg/kg; vit. B6 - 819mg/kg; vit. B12 – 4,150mcg/kg; vit. K3 – 537mg/kg; vit. D3 – 689UI/kg; calcium pantothenate – 3,230mg/kg; niacin – 9,800mg/kg; folic acid – 200mg/kg; biotin – 20mg/kg; zinc – 12g/kg; iron – 12g/kg; manganese – 14g/kg; copper – 3,120mg/kg; iodine – 252mg/kg; cobalt – 76mg/kg; selenium – 75mg/kg; ethoxyquin – 52mg/kg; B.H.A. – 40mg/kg; Q.S.P. vehicle – 1,000mg/kg.
